# Spatial structure of reproductive success infers mechanisms of ungulate invasion in Nearctic boreal landscapes

**DOI:** 10.1002/ece3.7103

**Published:** 2020-12-17

**Authors:** Jason T. Fisher, A. Cole Burton

**Affiliations:** ^1^ School of Environmental Studies University of Victoria Victoria BC Canada; ^2^ InnoTech Alberta Vegreville AB Canada; ^3^ Faculty of Forestry University of British Columbia Vancouver BC Canada

**Keywords:** camera trapping, fitness, invasive species, landscape change, multistate occupancy models, range shifts, reproduction

## Abstract

1. Landscape change is a key driver of biodiversity declines due to habitat loss and fragmentation, but spatially shifting resources can also facilitate range expansion and invasion. Invasive populations are reproductively successful, and landscape change may buoy this success.
2. We show how modeling the spatial structure of reproductive success can elucidate the mechanisms of range shifts and sustained invasions for mammalian species with attendant young. We use an example of white‐tailed deer (deer; *Odocoileus virginianus*) expansion in the Nearctic boreal forest, a North American phenomenon implicated in severe declines of threatened woodland caribou (*Rangifer tarandus*).
3. We hypothesized that deer reproductive success is linked to forage subsidies provided by extensive landscape change *via* resource extraction. We measured deer occurrence using data from 62 camera traps in northern Alberta, Canada, over three years. We weighed support for multiple competing hypotheses about deer reproductive success using multistate occupancy models and generalized linear models in an AIC‐based model selection framework.
4. Spatial patterns of reproductive success were best explained by features associated with petroleum exploration and extraction, which offer early‐seral vegetation resource subsidies. Effect sizes of anthropogenic features eclipsed natural heterogeneity by two orders of magnitude. We conclude that anthropogenic early‐seral forage subsidies support high springtime reproductive success, mitigating or exceeding winter losses, maintaining populations.
5. *Synthesis and Applications*. Modeling spatial structuring in reproductive success can become a key goal of remote camera‐based global networks, yielding ecological insights into mechanisms of invasion and range shifts to inform effective decision‐making for global biodiversity conservation.

## Graphical abstract

White‐tailed deer are expanding into the boreal with detrimental effects on the mammal community. Landscape change via resource extraction best explains successful deer reproduction in this boreal landscape.

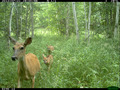

## 1 INTRODUCTION

Reproduction is vital to population persistence and distribution dynamics. Reproductive success is tightly linked to the quality and spatial distribution of available suitable habitat (Kurki et al., 2000; Pulliam & Danielson, 1991); an animal's habitat selection within its home range affects lifetime reproductive success (McLoughlin et al., 2007), and so, anthropogenic landscape change can markedly alter a population's spatial distribution. Anthropogenic disturbance is typically negative, through fragmentation and habitat loss (Fahrig, 1997, 2002, 2003), but is positive for some species, facilitating range expansions or invasions (Didham et al., 2007; Ewers & Didham, 2006). Linking spatial variability in reproductive success with landscape change is key to understanding mechanisms of invasion and range shifts, an increasingly important endeavor under climate change (Lawler et al., 2008, 2009).

Quantifying spatial variation in reproductive success has been mostly limited to taxa with stationary offspring such as plants (Muñoz & Arroyo, 2006) and nesting birds (León‐Ortega et al., 2017; Rosenberg et al., 2003). Quantifying this variation in mammals is much more difficult due to their large size, widespread ranges, and vagile young. Camera trapping (Burton et al., 2015; Steenweg et al., 2016) can bridge this data gap, generating data on mammalian distribution and density. Many mammal species keep young at heel during early maternal care, and this state can be observed with camera traps. In previous works on grizzly bears (Fisher et al., 2014) and European brown bears (Burton et al., 2018), we showed how spatial variation in reproductive success can be modeled to identify landscape mechanisms affecting success. Though diverse opportunities exist for multistate occupancy models to inform ecology and conservation (MacKenzie et al., 2017) their application to camera data have yet to be widely realized. Here, we illustrate how camera‐trap data can help infer mechanisms of species invasion and range expansion, using an example from the Nearctic boreal forest.

Boreal landscapes have been markedly changed by widespread and economically important resource extraction (Schindler & Lee, 2010; Venier et al., 2014). The epicenter of change are Canada's oil sands, the third largest global oil deposit and a driver of global economies (Bayoumi & Mhleisen, 2006). Petroleum exploration and extraction create an altered landscape without analogs (Pickell et al., 2013, 2015; Schneider et al., 2006). Landscape change affects the entire boreal forest mammal community (Fisher & Burton, 2018), but most notably manifests in woodland caribou declines (*Rangifer tarandus*) (Hebblewhite, 2017; Hervieux et al., 2013). Wolf (*Canis lupus*) predation is a primary cause (Boutin et al., 2012), with wolf populations bolstered by high‐density invading white‐tailed deer (deer; *Odocoileus virginianus*) (Latham et al., 2011, 2013).

White‐tailed deer range expansion is a pan‐continental phenomenon (Heffelfinger, 2011; Laliberte & Ripple, 2004) impacting entire ecosystems (Côté et al., 2004). Research on deer expansion south of the boreal has focused on population biology (DeYoung, 2011), movement (Beier & McCullough, 1990), and predation (Ballard et al., 2001). Large‐scale patterns of boreal deer invasion have been linked to climate change (Dawe et al., 2014; Dawe & Boutin, 2016) and landscape change (Fisher & Burton, 2018; Fisher et al., 2020), but the mechanisms remain unidentified. We sought to examine whether anthropogenic landscape change is linked to spatial patterns of deer reproductive success, as a possible mechanism of boreal forest invasion.

Deer obtain energy from early‐seral deciduous forage (Ditchkoff, 2011); intake must exceed metabolic demands which are markedly increased by cold temperatures and deep snow, and which have historically limited white‐tailed deer range (Hewitt, 2011; Parker et al., 2009). In the boreal, climate change has produced warmer winters (Karl & Trenberth, 2003); concurrently, landscape change has generated more abundant early‐successional vegetation (Finnegan et al., 2018, 2019; MacDonald et al., 2020) that is strongly spatially linked to deer abundance and persistence (Fisher et al., 2020). Deer mortality risk is greatest in the first year of life (Lesage et al., 2001), decreasing markedly for 1‐ to 2‐year‐olds (Delgiudice et al., 2006). Fawn growth and survival are largely based on maternal body condition, governed by food availability (Therrien et al., 2008), so examining how spatial resource availability contributes to breeding success within the first year helps us understand how landscape change contributes to boreal deer expansion.

We hypothesized that anthropogenic landscape change in the northern boreal forest is providing resource subsidies that bolster reproductive success for invading white‐tailed deer. If true, we predicted that anthropogenic features representing conversion of mature forest to early‐seral vegetation would explain variability in the spatial distribution of deer reproductive success. We define *reproductive success* as a deer occurrence with at least one attendant fawn in the summer months. This measure of reproductive success requires that a female achieve estrus, breed, produce offspring, and maintain that offspring into the summer months, thus drawing close to recruitment. It is a measure that can be consistently applied to all mammal species with attendant young at heel.

## 2 METHODS

### 2.1 Study area

**Figure 1:**
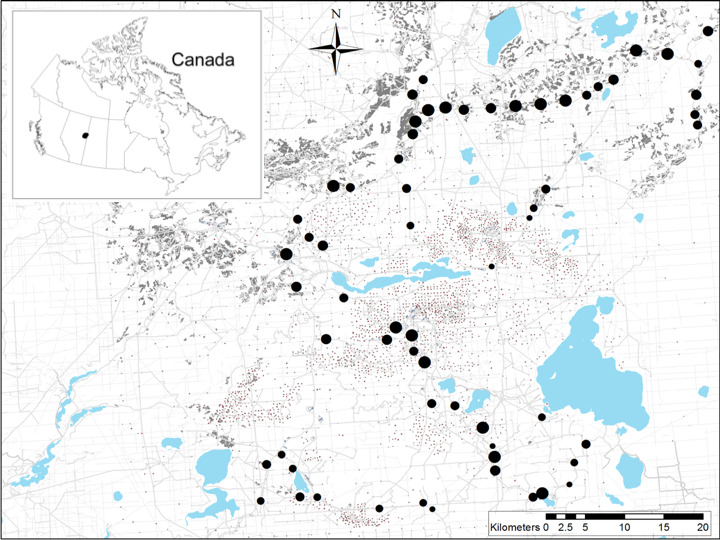
Occurrence of white‐tailed deer was surveyed at 62 camera sites (large block dots, scaled to deer relative abundance) in the boreal forest of northeast Alberta, Canada. Anthropogenic landscape features are widespread across this landscape, including forest harvesting cutblocks (gray polygons), well sites (square dots), seismic lines (gray), and roads and trails (dark gray and colored lines). Lakes are in blue

We surveyed white‐tailed deer distribution in the boreal forest of northeast Alberta, Canada (Figure 1). The 3,500 km^2^ landscape is a mosaic of aspen (*Populus tremulodies*), white spruce (*Picea glauca*), black spruce (*P. mariana*), and jack pine (*Pinus banksiana*) forests, interspersed with *Ledum groenlandicum*‐dominated muskeg. Widespread petroleum exploration and extraction features, roads (car accessible), trails (off‐road vehicle accessible), forest harvesting, and other anthropogenic features are dispersed throughout the study area (Figure 1).

We deployed 62 camera‐trap sites (Reconyx PC900 Hyperfire™ infrared remote digital; Holmen, WI, USA) in a constrained stratified random design (Supplementary Information), sampled continuously between November 2011 and November 2014, as in Fisher and Burton (2018), and Fisher et al. (2020). Following Burton et al. (2015), we define “site” as the average area used by a deer (seasonally, in a 3‐month window), centered on the camera detection zone. We define “study area” as the *ca*. 3,500 km^2^ minimum convex polygon surrounding camera sites. Cameras were placed *ca*. 1 m from the ground facing the wildlife trail and set to high sensitivity with 3‐s delay.

### 2.2 Spatial reproductive success

**Figure 2:**
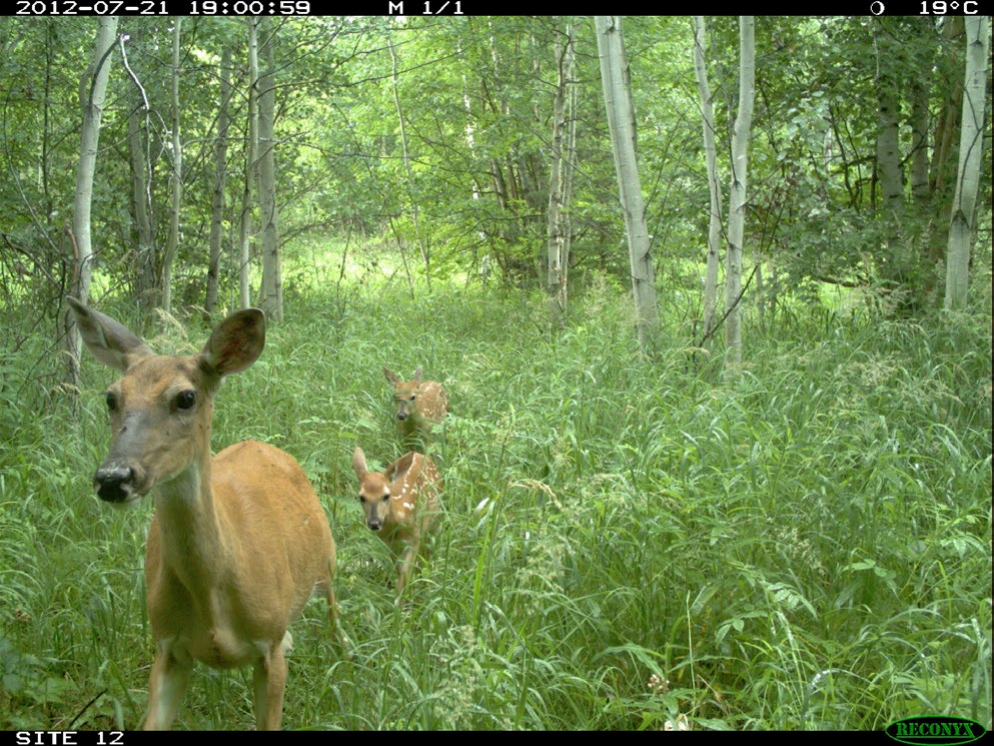
In the boreal forest of Alberta, Canada, camera traps quantified sites with white‐tailed deer fawns—characterized by their small size, and for younger animals, the presence of spots. Sites with fawns appearing in a survey month were recorded as “fawning” for that month

We identified all camera‐trap images containing white‐tailed deer and created a monthly detection–nondetection dataset with three states: breeding (fawns present in spring; hereafter “fawning”), nonbreeding, or no deer detected. We discretized continuous camera sampling into monthly survey occasions. If a fawn(s) appeared in an image within the survey month, we classified that site as “breeding” for that survey (Figure 2). If fawns were not detected, we classified the site as “nonbreeding”—which includes males and/or females that did not successfully rear a fawn into spring and summer.

Multiple approaches are used for modeling serial occurrence data generated by camera traps, depending on assumptions about detectability (Banks‐Leite et al., 2014; Burton et al., 2015; Rota et al., 2009). We analyzed camera data using two approaches. First, we sought to account for false absences—a potential problem in wildlife surveys (MacKenzie, 2005), including camera‐trap surveys (Burton et al., 2015). Just as species may be detected imperfectly, age–sex classes may also be detected imperfectly, when neither age nor sex is known with accuracy. In our case, “breeding” sites could be misclassified as “nonbreeding” if we missed photographing extant fawns at the cameras. To account for this possible error, we used occupancy models (MacKenzie et al., 2002) which estimate the probability of detecting that species if present (*p*) and based on *p*, the probability of site occupancy (*ψ*). With hierarchical multistate occupancy models (Nichols et al. 2007; MacKenzie et al., 2009), we estimated the probability for each site that deer were either absent, present without breeding, or present with breeding. We also estimated the probability that deer were detected in each of the two occupied states. Occupancy models can be considered as simultaneous generalized linear models (GLMs) applied to the detection and occupancy submodels, with binomial errors (logistic link).

We separated continuous camera data into month‐long (30.4 days) “secondary” survey periods sensu MacKenzie et al. (2003). Three such surveys comprised a three‐month “primary” sampling season within which occupancy states were assumed to be closed. We considered only the fawning (spring, April–June) and postfawning (summer, July–September) seasons. We assumed non‐Markovian variation in deer site use among months within a 3‐month season primary season (MacKenzie et al., 2006). In an occupancy framework, this variation represents “detection error,” attributed mainly to movement in and out of the camera detection zone (Burton et al., 2015). The full data frame for the study is thus 2 seasons per year × 3 years = 6 seasons, each season having 3 monthly surveys, totaling 18 surveys per site. In summary, the response variable for occupancy analyses is the number of months of deer detected (or not), and number of months of deer with fawns detected (or not), generating a serial detection dataset of 1 s and 0 s for each site.

With this dataset, we fit several competing models, each with different assumptions about how detectability, breeding occupancy, and nonbreeding occupancy varied through time and in relation to landscape features. We tested whether the probability of detection was either (1) constant over time, (2) varied among seasons, or (3) varied among surveys. We likewise tested whether site occupancy of breeders and nonbreeders was either (1) constant across the study area or (2) varied in relation to landscape features (Table S2). We used hierarchical models in the program *Presence* (ver. 6.2) to estimate deer occupancy (*ψ*), detectability (*p*), and breeding state (*R*), where:

*ψ_i_* = probability that site *i* is occupied, regardless of reproductive state
*R_i_* = conditional probability that breeding occurred, given that site *i* is occupied
*ψ_i_*_(_ *_b_*_)_ = unconditional probability that site *i* is occupied with breeding = *ψ_i_* * *R_i_*
*p*(1)*_it_* = probability that occupancy is detected for site *i*, period *t*, given that true state = 1 (nonbreeding)
*p*(2)*_it_* = probability that occupancy is detected for site *i*, period *t*, given that true state = 2 (breeding)
*δ_it_* = probability that evidence of successful reproduction is found, given detection of occupancy at site *i*, period *t*, with successful reproduction (Nichols et al., 2007).

Occupancy models provide a per‐survey estimate of *p*, and from this, we calculated the probability of false absence (PFA) across the three surveys in each sampling season as [1 − *p*]^3^ (Long et al., 2008).

In our second approach, we acknowledge that variation in detection–nondetection among secondary surveys (months of detections) can be due to animal movement and temporally variable habitat use; as such, it is an important part of the ecological signal, and not error as assumed in occupancy models (Broadley et al., 2019; Neilson et al., 2018; Stewart et al., 2018b). We therefore also treated zeros as signal, not error, and used an alternative modeling approach—generalized linear models (GLMs)—to determine whether fawn occurrence varied with landscape features. In this analysis, each month can be considered an independent Bernoulli trial in which adult female deer with fawns were detected (1) or not (0) (Faraway, 2016). We summed the number of spring months (April, May, June) with and without fawns across all three survey years. Here, the response variable is a number of months of deer with fawns detected (1) or not (0), ranging from 0 to 9 months (3 spring months over 3 years). We modeled the number of months in which fawns were observed using a binomial count model (GLM; binomial errors, log link) in R ver. 3.1.1 (R Foundation for Statistical Computing, 2014) against explanatory variables from three spatial digital resource inventories (Table S1).

In summary, we applied the same model selection approach to two model constructs—an occupancy model that assumes 1 s are detections and 0 s are missed detections, and a GLM approach that assumes 1 s are presences and 0 s are absences—and looked for concordance and departure among them (Banks‐Leite et al., 2014; Stewart et al., 2018a).

### 2.3 Landscape data

Alberta Vegetation Inventory (AVI), a digital forest inventory dataset, provided percent cover of landcover types within a 1‐km radius around each camera site (Fisher et al., 2011, 2020). Alberta Biodiversity Monitoring Institute (ABMI) 2010 Human Footprint Map Ver 1.1 provided percent of area of polygonal anthropogenic features. ABMI's Caribou Monitoring Unit (CMU) provided a GIS layer derived from 2012 SPOT satellite imagery to calculate area of linear features (buffered to create polygons from polylines) around each camera. Spatial data remained fixed during the three years of study. In all models, we omitted correlated variables (*r* > 0.7) from multiple‐variable models (Zuur et al., 2010) to prevent multicollinearity. We combined similar variables only sparsely represented in the data (<1%–2% of area) into a single, combination variable (Table 1), and rescaled each variable (mean = 0, *SD* = 1) to compare effect sizes.

**Table 1:** Hypotheses about the distribution of fawning white‐tailed deer across the boreal forest study area

Description	Model #	Hypothesis: White‐tailed deer distribution is explained by % cover (within a buffer around camera sites) of:
Global model	1	All variables
Natural landcover	2	Upland deciduous cover
	3	All mixedwood cover
	4	All conifer cover
	5	Upland spruce
	6	All deciduous + shrubs
	7	Wetland
	8	Upland forest
	9	Lowland forest
Nonforest	10	Early seral
Forestry	11	Cutblocks
Petroleum	12	Well sites
	13	3D seismic lines—narrow, high‐density lines in hashtags
	14	Seismic lines—wider, single strips
	15	Pipelines
	16	3D seismic lines + seismic lines + pipelines
	17	Well sites + industrial block features + nonforest
Petroleum + Forestry	18	Well sites + industrial block features + nonforest _ cutblocks
Access	19	Roads
	20	Trails
	21	Roads + trails
All anthropogenic	22	Models 16 + 18 + 22
Natural + forestry	23	Upland deciduous + cutblocks
	24	Shrubs + cutblocks
	25	Nonforest + cutblocks
Natural + petroleum	26	Upland deciduous + 3D seismic
	27	Upland deciduous + Seismic lines
	28	Upland deciduous + Model 22
	29	Upland deciduous + 16 + 17
Natural + access	30	Upland deciduous + roads + trails
*Post hoc* stepAIC model	31	Variables selected by stepwise regression

In occupancy models, we modeled *ψ* and *R* as a function of covariates or estimated each as a single parameter with no variation (Table S2). In GLMs, we created multiple a priori models, each corresponding to a hypothesis about the landscape features explaining variation in deer reproduction (Table 1). As a priori models may still contain uninformative parameters that should be discarded (Anderson, 2007), we additionally created a fully reduced model using AIC‐based stepwise regression (R; *stepAIC* package) to determine the most parsimonious model explaining variation in deer reproduction.

For both the occupancy models and GLMs, we weighed the evidence in support of models corresponding to competing hypotheses using model selection in an information‐theoretic framework (Burnham & Anderson, 2002). Each model produces an Akaike information criterion (AIC) score that balances deviance explained by the model with model complexity—the number of parameters; low AIC scores suggest a best‐supported model. We normalized AIC scores into 0–1 AIC weights, analogous to the probability that a given model is the best supported of the candidate set (Burnham & Anderson, 2002). We further validated best‐supported models using k‐fold cross‐validation in R package *boot* and calculated deviance explained.

## 3 RESULTS

### 3.1 Evidence of fawns

Of 112,648 deer images captured during the survey, 12,460 images (11.1%) had evidence of young of the year. This included single fawns (92.9%), twins (7.6%), and triplets (0.6%), though these were not distinguished in models. There was a marked drop in the distribution of deer with fawns across years. Of 62 sites, fawns were detected at 36 sites (58.1%) in 2012, 22 (35.5%) in 2013, and 12 (19.4%) in 2014. These represent naïve observations, unadjusted for probability of detection. Among all years pooled together, 45 of 62 sites (72.6%) had evidence of deer with fawns in at least one year.

### 3.2 Multistate deer occupancy

Accounting for probability of detection, reproductively successful deer—does with fawns—were estimated to be widespread across the study area in spring 2012 (*ψ_b_* = 0.89, *SE* = 0.14), 2013 (*ψ_b_* = 0.98; *SE* = 0.02), and 2014 (*ψ_b_* = 0.95; *SE* = 0.03) when modeled without landscape covariates. PFA of deer with fawns [1 − *p*]^3^ was ≤0.002 in all years, suggesting that we reliably detected fawns when they occurred.

Anthropogenic landscape features best explained conditional probability of fawns given occupancy by deer (*R*) in 2012 (well sites and seismic lines, cumulative AIC_w_ = 0.83), 2013 (seismic lines, AIC_w_ = 0.84), and 2014 (industrial features, forest cutblocks, and total footprint, cumulative AIC_w_ = 0.81) (Table S2). Models in which fawn occurrence varied only with natural vegetation, or was invariant, were not supported. Hence, occupancy of deer with fawns differed from that of deer without fawns and varied with the area of anthropogenic features across the oil sands landscape. However, some multistate occupancy models contained unresolvable “border estimates” for *R*, a noted problem when estimates approach 0 or 1 (MacKenzie et al., 2017).

### 3.3 Spatial patterns of reproductive success: GLMs

**Figure 3:**
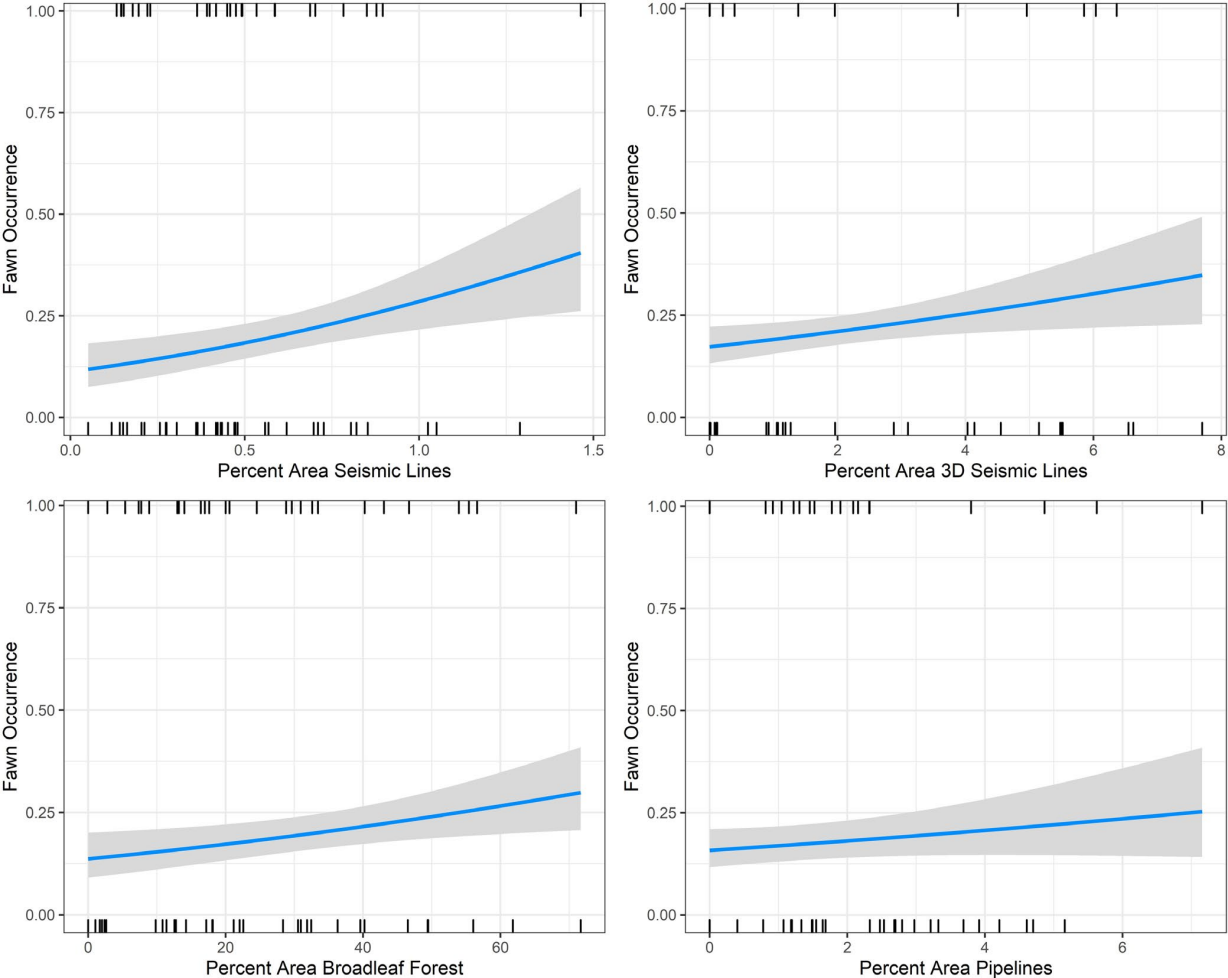
Spatial variation in white‐tailed deer reproductive success in the oil sands of the western Nearctic boreal forest of Alberta, Canada, was best explained by the post hoc *stepAIC* model (#31) which included petroleum extraction features—conventional seismic lines, 3D seismic lines, and pipelines—as well as upland deciduous forest. Gray shading represents 95% confidence intervals. Abscissae are scaled to the range maximum for that variable

**Figure 4:**
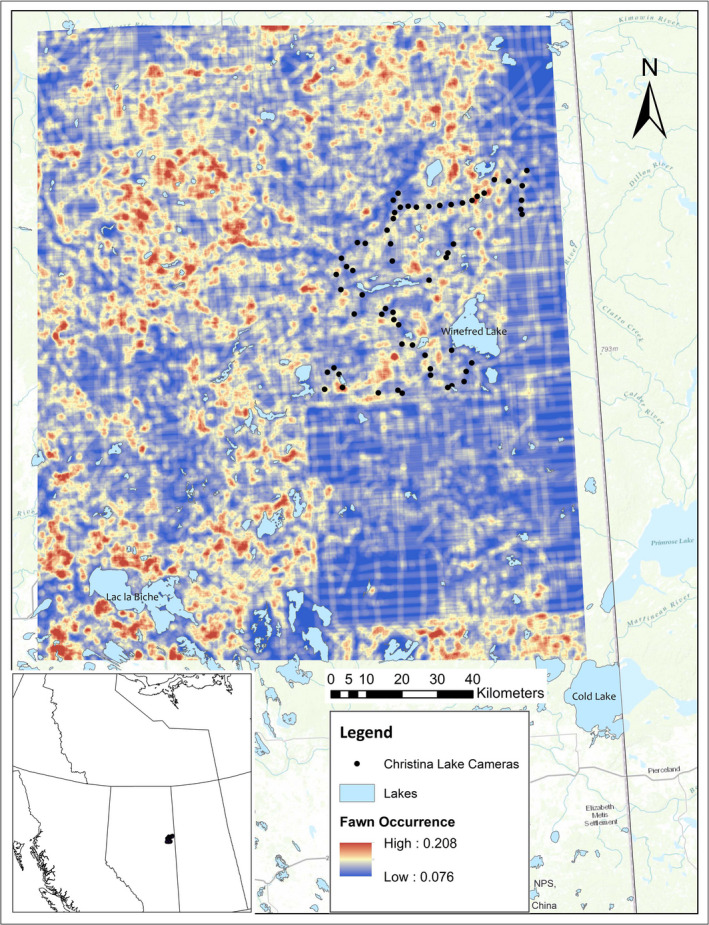
The probability of white‐tailed deer reproduction across Alberta's northeast boreal forest oil sands' region. Beta coefficients from the best‐supported generalized model explaining spatial variation in fawn occurrence were extrapolated across the region using the same spatial data from which the models were derived

Months of occurrence of reproductively successful deer were positively related to anthropogenic landscape features, as well as natural landscape features. Models with petroleum features best explained deer with fawn occurrence, thus corroborating the multistate occupancy models. Occurrence of deer with fawns increased with increasing seismic line density (long, wide cleared strips), 3D seismic line density (short, narrow, strips crisscrossed in high density with a hashtag shape), pipeline density, and deciduous forest cover; the *post hoc stepAIC* model (#31; AIC_w_ = 0.88) and the *upland deciduous and all petroleum features* model (#29, AIC_w_ = 0.11) together carried 99% of the weight of evidence (Table 2; Table S3). The effect size (model β‐coefficients) of seismic lines on the occurrence of fawns was 100 times greater than the effect size of the best natural landcover feature: upland deciduous forests (Figure 3). Probability of fawn occurrence more than tripled from ~0% seismic line density to only 1.5% seismic line density. In comparison, probability of fawn occurrence roughly doubles from 0% broadleaf forest to 80% broadleaf forest. Projected across the northeast boreal forest surrounding the study area, areas of higher probability of deer reproduction correspond to intensive development (Figure 4).

**Table 2:** Model selection of generalized linear models relating probability of occurrence of white‐tailed deer with fawn(s) against natural and anthropogenic landscape features

Model #	K	AIC	ΔAIC	AIC_w_	Cumulative AIC_w_	−2LL
Model 31	5	406.41	0	0.88	0.88	−197.66
Model 29	7	410.64	4.23	0.11	0.99	−197.26
Model 28	11	415.69	9.28	0.01	1	−194.15
Model 27	4	428.09	21.68	0	1	−209.69
Model 16	7	430.79	24.38	0	1	−207.34
Model 22	10	431.07	24.67	0	1	−203.34
Model 14	3	433.95	27.54	0	1	−213.77
Model 1	11	438.14	31.73	0	1	−205.38
Model 4	6	438.53	32.12	0	1	−212.49
Model 7	4	441.22	34.81	0	1	−216.25
Model 6	6	441.28	34.87	0	1	−213.86
Model 26	4	443.1	36.7	0	1	−217.19
Model 23	4	443.16	36.76	0	1	−217.23
Model 30	5	443.69	37.28	0	1	−216.3
Model 2	3	444.64	38.23	0	1	−219.11
Model 8	5	446.73	40.32	0	1	−217.82
Model 15	3	447.25	40.84	0	1	−220.41
Model 17	4	451.05	44.65	0	1	−221.17
Model 10	7	452.6	46.19	0	1	−218.24
Model 18	5	452.79	46.38	0	1	−220.85
Model 20	3	452.86	46.45	0	1	−223.22
Model 12	3	453.11	46.7	0	1	−223.34
Model 5	3	453.16	46.75	0	1	−223.37
Model 21	4	454.68	48.27	0	1	−222.98
Model 19	3	454.7	48.3	0	1	−224.14
Model 9	5	454.79	48.39	0	1	−221.85
Model 13	3	454.8	48.39	0	1	−224.19
Model 11	3	455.07	48.67	0	1	−224.33
Model 3	4	455.97	49.56	0	1	−223.63
Model 25	5	456.41	50	0	1	−222.66
Model 24	4	457.37	50.96	0	1	−224.33

Note

Model numbers refer to candidate model sets in Table 2. K = number parameters.

## 4 DISCUSSION

As mammal distributions shift with climate change, decrease with habitat loss, or capitalize upon change to invade, understanding the features facilitating species' reproduction in once unsuitable landscapes allows us to elucidate, and address, those mechanisms of change. Global camera‐trap networks coupled with species distribution models can yield these insights.

In our example, widespread landscape change from energy extraction is strongly linked to white‐tailed deer reproduction where they have invaded the western Nearctic boreal forest. Deer invasion of the cold northern latitudes is consequent to expansion from southern agricultural areas, a continental phenomenon borne from widespread conversion of mature forest into early‐seral vegetation (Côté et al., 2004; Heffelfinger, 2011). In the last few decades, new advances in forest harvesting and the marked growth of energy exploration and extraction have substantially altered the Alberta boreal landscape (Pickell et al., 2013, 2015). The density of petroleum exploration seismic lines in the landscape had a 100 times greater effect on probability of fawns occurring than did natural deciduous forest, an important predictor of adult white‐tailed deer individual habitat selection and distribution (Darlington, 2018; Fisher & Burton, 2018; Fisher et al., 2020). Although we hypothesized that forest harvesting might play a substantial role (Fisher & Wilkinson, 2005), we found no evidence to support this, and petroleum extraction features are much more widespread than forest harvest blocks in this region (Pickell et al., 2013, 2015). We conclude that the 1,000s of kilometers of seismic lines, as well as pipelines and 3D seismic lines, spread across the western Nearctic boreal forest play a significant role in facilitating the northward expansion of white‐tailed deer.

The mechanism for the relationship between linear features and deer reproductive success is centered on available forage. Nutrition affects ungulates' probability of pregnancy, over‐winter survival, parturition, and neonatal survival (Hewitt, 2011; Parker et al., 2009). Greater nutrition from abundant available forage prevents metabolic stress, increasing deer survivorship and reproductive success (Hewitt, 2011). However, forage biomass is in itself not a good predictor of deer nutrition, as forage distribution relative to inedible vegetation plays a significant role (Spalinger & Hobbs, 1992). In this landscape, abundant edible forage is available in linear features (Finnegan et al., 2018, 2019; MacDonald et al., 2020), and may be especially important in spring during green‐up, when energetic demands of gestation are great (Pekins et al., 1998).

Research on deer pregnancy rates and recruitment suggests that female age and body condition affect breeding success (DelGiudice et al., 2007; Ozoga & Verme, 1986; Ozoga et al., 1982; Verme, 1989); body condition, in turn, is primarily a function of nutrition afforded by available browse (Hewitt, 2011). Winter induces substantial metabolic costs on white‐tailed deer, but pregnancy and lactation induce markedly greater metabolic costs on females (Ditchkoff, 2011; Pekins et al., 1998; Therrien et al., 2008). If female deer in this landscape were metabolically stressed after severe winters, female mortality, small fawns with low survival (Ditchkoff, 2011), and starvation‐induced abortions (Worden 1992, in Pekins et al., 1998) might be expected to reduce reproductive success. If the early‐seral vegetation abundant in anthropogenic landscape features provides forage subsidies, then metabolic costs would be offset resulting in reproductive success. We contend our evidence here, as well as corroborating past research on adult deer showing positive links to anthropogenic features (Darlington, 2018; Fisher & Burton, 2018; Fisher et al., 2020), strongly infers that landscape change is enhancing recruitment and hence, facilitating and maintaining boreal deer invasions.

### 4.1 Caveats

Our observations might be thought to be due to differential habitat selection among sexes. We could not reliably tell males from females with certainty, in the autumn when adult males have pronounced antlers it is possible, but not annually and not for young males. When we could discern sexes, we did not observe substantial sexual segregation, nor is prolonged sexual segregation of white‐tailed deer suggested in the literature, so we attribute our observations to differential fawning success.

Our research focused on a heavily developed landscape in the western Nearctic boreal forest of Alberta, Canada. Extrapolating to other landscapes in this region should not be done without future research to understand the range of inference. In their province‐wide analysis, Dawe et al. (2014), and Dawe and Boutin (2016) concluded that deer expansion is likely facilitated in large part by climate change as the metabolic costs of cold temperatures and especially deep snow are ameliorated by contemporary mild winters. Evidence at landscape scales suggests climate is a contributory mechanism but abundant nutritional forage is pivotal for deer populations (Fisher et al., 2020), and historically, the northern boreal forest has been dominated by largely inedible conifer (Fisher & Wilkinson, 2005; Pickell et al., 2015). We contend forage subsidies induced by landscape change play a large role not yet disentangled from climate change; indeed, it is likely the two act synergistically.

## 5 APPLICATIONS TO ECOLOGY

In the western boreal forest, petroleum exploration features are increasing deer fawning success and hence possibly (given lifetime success) fitness of individuals spatially associating with them. In the apparent competition “fulcrum” in which more deer boost wolf populations, which in turn drive declines in woodland caribou (Boutin et al., 2012; DeCesare et al., 2010; Latham et al., 2011), deer expansion is a substantial conservation threat. Conservation will require landscape management to mitigate the widespread resource subsidies afforded to deer, including active site restoration, which has been shown to be promising for mitigating white‐tailed deer use of seismic lines (Tattersall et al., 2019). Dauntingly, this restoration is required for 10,000s of kilometers of seismic lines (Dabros et al., 2018), as well as the other anthropogenic features associated with resource extraction (Fisher & Burton, 2018; Fisher et al., 2020) lending urgency to the need for rapid application of ecological research to management decisions.

Biodiversity declines due to landscape change are a global problem (Maxwell et al., 2016) as are invasive species (Clavero & García‐Berthou, 2005; Gurevitch & Padilla, 2004) and anthropogenic range shifts (Chen et al., 2011; Lawler et al., 2009). Understanding the ecological mechanisms facilitating and sustaining invasions is a key pursuit for and ecology. Global biodiversity networks can quantify variation in mammalian distribution and density at large scales (Steenweg et al., 2016), but abundance is not always a reliable metric for inference of mechanisms (Battin, 2004; Schlaepfer et al., 2002; Van Horne, 1983). Reproductive success is more directly reflective of landscape change's effect on mammalian fitness. These data can be garnered through camera‐trap networks and modeled with data on landscape change to aid inference about the mechanisms of change: an intersection of fundamental ecology principles and applied ecology practice that can aid inferences and the decisions derived from them.

## CONFLICT OF INTEREST

The authors have no competing interests or conflict of interest to declare.

## AUTHOR CONTRIBUTIONS

**Jason T. Fisher:** Conceptualization (lead); data curation (lead); formal analysis (lead); funding acquisition (lead); investigation (lead); methodology (lead); project administration (lead); writing‐original draft (lead); writing‐review & editing (equal). **A. Cole Burton:** Conceptualization (equal); data curation (supporting); formal analysis (supporting); funding acquisition (supporting); investigation (supporting); methodology (supporting); validation (lead); writing‐review & editing (supporting).

## RESEARCH ETHICS

All research was permitted by the Government of Alberta, Ministry of Environment and Parks, Fish & Wildlife Division, Collection License 49143.

## ANIMAL ETHICS

This research was reviewed and approved by the InnoTech Alberta's Animal Care and Use Committee (ACUC), permit ACUC0524.frm /clj/IO.II.02.

## Supporting information

Supplementary Material

DeerReproSuccesscode

DeeeproSuccess

## Data Availability

Data and R code used in these analyses are available on Dryad at https://doi.org/10.5061/dryad.xksn02vf2.
