# Supplementary material for: Spatial structure of reproductive success infers mechanisms of ungulate invasion in Nearctic boreal landscapes
Source: Ecol Evol. 2020 Dec 17;11(2):900–11. doi: 10.1002/ece3.7103 (PMC7820139; doi:10.1002/ece3.7103)
Supplement: Supplementary file 1 — Supplementary Material [file ECE3-11-900-s001.docx]

Spatial structure of reproductive success infers mechanisms of ungulate invasion in Nearctic boreal landscapes.

Jason T Fisher^1,2, *^ and A. Cole Burton^3,2^

^1^University of Victoria, School of Environmental Studies, Victoria, British Columbia, Canada

^2^Former Address: InnoTech Alberta, Bag 4000, Vegreville, Alberta, Canada

^3^University of British Columbia, Faculty of Forestry, Vancouver, British Columbia, Canada

*Corresponding author: fisherj@uvic.ca

**Supplementary Information**

**Methods**

We reclassified the landscape into landcover categories (Table S1) using GIS. We overlaid these data with 1x1-km grid cells, categorized each by dominant landcover and then randomly selected grid cells so that each landcover category received equal sampling effort. Random selection was constrained to induce a minimum 2-km distance from other sites to maintain independence (based on deer home range sizes; ([DeYoung 2011](#_ENREF_22); [Heffelfinger 2011](#_ENREF_38)). The design produced some sampling site clustering (Fig. 1) due to the naturally clumped distribution of forest types on the landscape but captured the range of this landscape's anthropogenic footprint and natural heterogeneity. Cameras were accessed *via* roads and trails, but the area is so replete with these features (Fig. 1) that beyond a 250-m radius around each camera roads are sampled as represented in the landscape (see multiscale analysis in ([Fisher & Burton 2018](#_ENREF_32))). We obtained permission from government land officers and industry leaseholders to access all sampling areas.

**Table S1.** Landscape reclassification for species distribution models. GIS data from multiple sources were reclassified and combined to create 20 different landscape feature categories. We calculated the percent area of each category around each camera site, to create 20 independent variables.

| Landcover Set | Landcover Class | Source^1^ | Description^2,3^ |
| --- | --- | --- | --- |
| Natural landscape features | Upland deciduous | AVI | (Aw, Pb, Bw >=70% canopy), moisture = d or m |
|  | Lowland deciduous | AVI | (Aw, Pb, Bw >=70% canopy), moisture = w or a |
|  | Upland mixedwood | AVI | (40% -60%) canopy, moisture = d or m |
|  | Lowland mixedwood | AVI | (40% -60%) canopy, moisture = w or a |
|  | Upland spruce | AVI | (Sb, Sw, Fb >=70% canopy), moisture = d or m |
|  | Lowland spruce | AVI | (Sb,Sw,Fb >=70% canopy), moisture = w or a |
|  | Pine | AVI | All Pj (>=70%) |
|  | Tamarack | AVI | All Lt (>=70%) |
|  | Open wetland | AVI | <6% crown closure; moisture = w or a |
|  | Upland shrubs | AVI | >25% shrub cover; <6% tree cover; moisture = d or m |
|  | Water | AVI | Standing or flowing water |
|  | Nonforest | AVI | < 6% canopy |
| Anthropogenic landscape features | Cutblocks | ABMI | Forest harvested areas, which regrow trees, and are important for deer in early seral stages. |
|  | Industrial block features | ABMI | Borrow-Pits, Dugouts, Sumps, Industrial Sites, and Other Disturbed Vegetation not falling into other categories |
|  | Well sites | ABMI | Petroleum extraction well sites, disturbed forest with canopy removed, areas now grassy or shrubby. |
|  | 3D seismic lines | CMU | Intensive (hashmarked) seismic petroleum exploration line *ca.* 1-3 m wide. |
|  | Seismic lines | CMU | Traditional seismic petroleum exploration line *ca.* 7-10 m wide and typically very long. |
|  | Roads | CMU | All roads including One Lane Gravel, One Lane Paved, Two Lane Gravel, Two Lane Paved, Unimproved Roads |
|  | Pipelines | CMU | Petroleum pipelines |
|  | Trails | CMU | Trail + TruckTrail: Unimproved dirt track ca. 5-10 m wide navigable by off-highway vehicle, foot, or horseback, or by truck. |

^1^AVI – Alberta Vegetation Index; ABMI = Alberta Biodiversity Monitoring Institute Human Footprint Map Updated 2010 ; ABMI-CMU = ABMI Caribou Monitoring Unit Linear Features Map Updated 2012 ^2^Aw = aspen, Pb = poplar, Bw = white birch, Sb = black spruce Sw = white spruce, Fb = balsam fir, Pj = jack pine, Lt = tamarack/larch; d = dry, m = mesic, w = wet, a = aquatic

^3^Linear features were buffered for areal calculations as follows: Two Lane Undivided Paved Road: 9m; One Lane Undivided Paved Road: 6m; Rail Line: 5.5m; Rail Line- spur: 5.5m; Two Lane Gravel Road: 7m; One Lane Gravel Road: 5m; Driveway: 2m; Unimproved Road: 6m; Trail: 6m; Truck Trail: 6m; Electrical Transmission Line: 17m; Pipeline: 12m; 3D: 2m; Cutline: 2m.

**Results**

**Table S2:** Model selection of multi-state hierarchical models of white-tailed deer, with variable states of breeding or non-breeding. Occupancy (Ψ) was held constant (.) while conditional probability of state 2 (breeding), R, could vary with multiple anthropogenic feature types or was constant (.). Probability of detection for state 1 (nonbreeding) and 2 (breeding) could vary with sampling season (S) or was constant (.). The probability of detecting breeding of present (δ) was held constant. ML = model likelihood; k = number of parameters; -2LL = log likelihood of the model.

| Year | Model | AIC | ΔAIC | AIC_w_ | ML | K | -2LL |
| --- | --- | --- | --- | --- | --- | --- | --- |
| 2012 | psi,R(WELLSITE),p1(S),p2(S),dlta(.) | 270.91 | 0.00 | 0.45 | 1.00 | 10 | 250.91 |
|  | psi,R(CUTLINE),p1(S),p2(S),dlta(.) | 271.25 | 0.34 | 0.38 | 0.84 | 9 | 253.25 |
|  | psi,R,p1(S),p2(S),dlta(.) | 274.59 | 3.68 | 0.07 | 0.16 | 9 | 256.59 |
|  | psi,R(CUTBLOCKS),p1(S),p2(S),dlta(.) | 275.70 | 4.79 | 0.04 | 0.09 | 10 | 255.70 |
|  | psi,R(CUTLINE),p1(.),p2(.),dlta(.) | 278.18 | 7.27 | 0.01 | 0.03 | 5 | 268.18 |
|  | psi,R(BLOCK),p1(.),p2(.),dlta(.) | 278.22 | 7.31 | 0.01 | 0.03 | 6 | 266.22 |
|  | psi,R(WELLSITE),p1(.),p2(.),dlta(.) | 278.87 | 7.96 | 0.01 | 0.02 | 6 | 266.87 |
|  | psi,R(BLOCK),p1(S),p2(S),dlta(.) | 279.26 | 8.35 | 0.01 | 0.02 | 10 | 259.26 |
|  | psi,R(DECID),p1(S),p2(S),dlta(.) | 279.30 | 8.39 | 0.01 | 0.02 | 10 | 259.30 |
|  | psi,R(PIPELINE),p1(S),p2(S),dlta(.) | 281.32 | 10.41 | 0.00 | 0.01 | 10 | 261.32 |
|  | psi,R(LINEAR),p1(.),p2(.),dlta(.) | 281.70 | 10.79 | 0.00 | 0.00 | 6 | 269.70 |
|  | psi,R,p1(.),p2(.),dlta(.) | 281.87 | 10.96 | 0.00 | 0.00 | 5 | 271.87 |
|  | psi,R(FOOTPRINT),p1(S),p2(S),dlta(.) | 282.09 | 11.18 | 0.00 | 0.00 | 11 | 260.09 |
|  | psi,R(3DSeismic),p1(S),p2(S),dlta(.) | 282.15 | 11.24 | 0.00 | 0.00 | 10 | 262.15 |
|  | psi,R(LINEAR),p1(S),p2(S),dlta(.) | 282.51 | 11.60 | 0.00 | 0.00 | 10 | 262.51 |
|  | psi,R(PIPELINE),p1(.),p2(.),dlta(.) | 283.63 | 12.72 | 0.00 | 0.00 | 6 | 271.63 |
|  | psi,R(CUTBLOCKS),p1(.),p2(.),dlta(.) | 283.69 | 12.78 | 0.00 | 0.00 | 6 | 271.69 |
|  | psi,R(DECID),p1(.),p2(.),dlta(.) | 284.97 | 14.06 | 0.00 | 0.00 | 6 | 272.97 |
|  | psi,R(3DSeismic),p1(.),p2(.),dlta(.) | 286.23 | 15.32 | 0.00 | 0.00 | 6 | 274.23 |
|  | psi,R(GLOBAL),p1(S),p2(S),dlta(.) | 286.51 | 15.60 | 0.00 | 0.00 | 12 | 262.51 |
|  | psi,R(FOOTPRINT),p1(.),p2(.),dlta(.) | 288.57 | 17.66 | 0.00 | 0.00 | 7 | 274.57 |
|  | psi,R(GLOBAL),p1(.),p2(.),dlta(.) | 290.57 | 19.66 | 0.00 | 0.00 | 8 | 274.57 |
|  |  |  |  |  |  |  |  |
| Year | Model | AIC | ΔAIC | AIC_w_ | ML | K | -2LL |
| 2013 | psi,R(CUTLINE),p1(S),p2(S),dlta(.) | 254.21 | 0.00 | 0.84 | 1.00 | 10 | 234.21 |
|  | psi,R,p1(S),p2(S),dlta(.) | 260.85 | 6.64 | 0.03 | 0.04 | 9 | 242.85 |
|  | psi,R(LINEAR),p1(S),p2(S),dlta(.) | 261.34 | 7.13 | 0.02 | 0.03 | 10 | 241.34 |
|  | psi,R(CUTBLOCKS),p1(S),p2(S),dlta(.) | 261.43 | 7.22 | 0.02 | 0.03 | 10 | 241.43 |
|  | psi,R(WELLSITE),p1(S),p2(S),dlta(.) | 262.30 | 8.09 | 0.01 | 0.02 | 10 | 242.30 |
|  | psi,R(3DSeismic),p1(S),p2(S),dlta(.) | 262.54 | 8.33 | 0.01 | 0.02 | 10 | 242.54 |
|  | psi,R(FOOTPRINT),p1(S),p2(S),dlta(.) | 262.58 | 8.37 | 0.01 | 0.02 | 11 | 240.58 |
|  | psi,R(DECID),p1(S),p2(S),dlta(.) | 262.71 | 8.50 | 0.01 | 0.01 | 10 | 242.71 |
|  | psi,R(PIPELINE),p1(S),p2(S),dlta(.) | 262.94 | 8.73 | 0.01 | 0.01 | 10 | 242.94 |
|  | psi,R(GLOBAL),p1(.),p2(.),dlta(.) | 263.27 | 9.06 | 0.01 | 0.01 | 8 | 247.27 |
|  | psi,R(BLOCK),p1(S),p2(S),dlta(.) | 264.60 | 10.39 | 0.00 | 0.01 | 10 | 244.60 |
|  | psi,R(DECID),p1(.),p2(.),dlta(.) | 265.27 | 11.06 | 0.00 | 0.00 | 6 | 253.27 |
|  | psi,R(GLOBAL),p1(S),p2(S),dlta(.) | 266.61 | 12.40 | 0.00 | 0.00 | 12 | 242.61 |
|  | psi,R(FOOTPRINT),p1(.),p2(.),dlta(.) | 266.63 | 12.42 | 0.00 | 0.00 | 7 | 252.63 |
|  | psi,R(BLOCK),p1(.),p2(.),dlta(.) | 267.80 | 13.59 | 0.00 | 0.00 | 6 | 255.80 |
|  | psi,R(CUTLINE),p1(.),p2(.),dlta(.) | 269.23 | 15.02 | 0.00 | 0.00 | 6 | 257.23 |
|  | psi,R,p1(.),p2(.),dlta(.) | 270.91 | 16.70 | 0.00 | 0.00 | 5 | 260.91 |
|  | psi,R(LINEAR),p1(.),p2(.),dlta(.) | 270.99 | 16.78 | 0.00 | 0.00 | 6 | 258.99 |
|  | psi,R(3DSeismic),p1(.),p2(.),dlta(.) | 271.51 | 17.30 | 0.00 | 0.00 | 6 | 259.51 |
|  | psi,R(WELLSITE),p1(.),p2(.),dlta(.) | 271.56 | 17.35 | 0.00 | 0.00 | 6 | 259.56 |
|  | psi,R(CUTBLOCKS),p1(.),p2(.),dlta(.) | 271.58 | 17.37 | 0.00 | 0.00 | 6 | 259.58 |
|  | psi,R(PIPELINE),p1(.),p2(.),dlta(.) | 272.91 | 18.70 | 0.00 | 0.00 | 6 | 260.91 |
|  |  |  |  |  |  |  |  |
| Year | Model | AIC | ΔAIC | AIC_w_ | ML | K | -2LL |
| 2014 | psi,R(BLOCK),p1(S),p2(S),dlta(.) | 228.30 | 0.00 | 0.45 | 1.00 | 10 | 208.30 |
|  | psi,R(CUTBLOCKS),p1(S),p2(S),dlta(.) | 229.98 | 1.68 | 0.19 | 0.43 | 10 | 209.98 |
|  | psi,R(FOOTPRINT),p1(S),p2(S),dlta(.) | 230.25 | 1.95 | 0.17 | 0.38 | 11 | 208.25 |
|  | psi,R(GLOBAL),p1(S),p2(S),dlta(.) | 232.03 | 3.73 | 0.07 | 0.15 | 12 | 208.03 |
|  | psi,R(BLOCK),p1(.),p2(.),dlta(.) | 233.28 | 4.98 | 0.04 | 0.08 | 6 | 221.28 |
|  | psi,R,p1(S),p2(S),dlta(.) | 234.31 | 6.01 | 0.02 | 0.05 | 9 | 216.31 |
|  | psi,R(CUTLINE),p1(S),p2(S),dlta(.) | 234.76 | 6.46 | 0.02 | 0.04 | 10 | 214.76 |
|  | psi,R(LINEAR),p1(S),p2(S),dlta(.) | 235.46 | 7.16 | 0.01 | 0.03 | 10 | 215.46 |
|  | psi,R(DECID),p1(S),p2(S),dlta(.) | 235.53 | 7.23 | 0.01 | 0.03 | 10 | 215.53 |
|  | psi,R(WELLSITE),p1(S),p2(S),dlta(.) | 236.09 | 7.79 | 0.01 | 0.02 | 10 | 216.09 |
|  | psi,R(FOOTPRINT),p1(.),p2(.),dlta(.) | 238.94 | 10.64 | 0.00 | 0.00 | 7 | 224.94 |
|  | psi,R(3DSEISMIC),p1(S),p2(S),dlta(.) | 239.10 | 10.80 | 0.00 | 0.00 | 10 | 219.10 |
|  | psi,R(DECID),p1(.),p2(.),dlta(.) | 239.88 | 11.58 | 0.00 | 0.00 | 6 | 227.88 |
|  | psi,R(GLOBAL),p1(.),p2(.),dlta(.) | 240.72 | 12.42 | 0.00 | 0.00 | 8 | 224.72 |
|  | psi,R(PIPELINE),p1(S),p2(S),dlta(.) | 242.80 | 14.50 | 0.00 | 0.00 | 10 | 222.80 |
|  | psi,R,p1(.),p2(.),dlta(.) | 243.65 | 15.35 | 0.00 | 0.00 | 5 | 233.65 |
|  | psi,R(CUTLINE),p1(.),p2(.),dlta(.) | 243.99 | 15.69 | 0.00 | 0.00 | 6 | 231.99 |
|  | psi,R(LINEAR),p1(.),p2(.),dlta(.) | 244.36 | 16.06 | 0.00 | 0.00 | 6 | 232.36 |
|  | psi,R(CUTBLOCKS),p1(.),p2(.),dlta(.) | 244.65 | 16.35 | 0.00 | 0.00 | 6 | 232.65 |
|  | psi,R(PIPELINE),p1(.),p2(.),dlta(.) | 245.63 | 17.33 | 0.00 | 0.00 | 6 | 233.63 |
|  | psi,R(3DSEISMIC),p1(.),p2(.),dlta(.) | 246.93 | 18.63 | 0.00 | 0.00 | 6 | 234.93 |
|  | psi,R(WELLSITE),p1(.),p2(.),dlta(.) | 252.40 | 24.10 | 0.00 | 0.00 | 6 | 240.40 |

**Table S3:** Parameter estimates from the best supported candidate model (Model 31) of white-tailed deer fawn occurrence (number of spring / summer months of detections over 3 years), modelled against natural and anthropogenic landscape features.

| Parameter | Estimate | std. error | z-value | p-value |
| --- | --- | --- | --- | --- |
| Seismic lines | 1.15 | 0.20 | 5.66 | < 0.00 |
| 3D seismic lines | 0.10 | 0.03 | 3.47 | < 0.00 |
| Pipelines | 0.08 | 0.03 | 2.55 | 0.01 |
| Upland deciduous forest | 0.01 | 0.00 | 4.48 | < 0.00 |
| (Intercept) | -2.49 | 0.19 | -13.03 | < 0.00 |
